# Transcriptome sequence analysis and mining of SSRs in Jhar Ber (*Ziziphus nummularia* (Burm.f.) Wight & Arn) under drought stress

**DOI:** 10.1038/s41598-018-20548-1

**Published:** 2018-02-05

**Authors:** Radha Yadav, Showkat Ahmad Lone, Kishor Gaikwad, Nagendra Kumar Singh, Jasdeep Chatrath Padaria

**Affiliations:** 1 Biotechnology and Climate Change Group, National Research Centre on Plant Biotechnology (ICAR-NRCPB), New Delhi, 110012 India; 2 Present Address: Department of Agricultural Microbiology, Faculty of Agricultural Sciences, Aligarh Muslim University, Aligarh, 202002 India

## Abstract

*Ziziphus nummularia* (Burm.f.) Wight & Arn., a perennial shrub that thrives in the arid regions, is naturally tolerant to drought. However, there are limited studies on the genomics of drought tolerance in *Ziziphus* sp. In this study, RNA-sequencing of one month old seedlings treated with PEG 6000 was performed using Roche GS-FLX454 Titanium pyrosequencing. A total of 367,176 raw sequence reads were generated, and upon adapter trimming and quality filtration 351,872 reads were assembled *de novo* into 32,739 unigenes. Further characterization of the unigenes indicated that 73.25% had significant hits in the protein database. Kyoto encyclopedia of genes and genomes database (KEGG) identified 113 metabolic pathways from the obtained unigenes. A large number of drought-responsive genes were obtained and among them differential gene expression of 16 highly induced genes was validated by qRT-PCR analysis. To develop genic-markers, 3,425 simple sequence repeats (SSRs) were identified in 2,813 unigene sequences. The data generated shall serve as an important reservoir for the identification and characterization of drought stress responsive genes for development of drought tolerant crops.

## Introduction

Incidence of drought is one of the important causes responsible for reduction in crop yields that arises due to inadequate and unevenly distributed rainfall. Plants respond to drought stress by activating an intricate network of protein-coding genes involving various biochemical and molecular mechanisms^1,2^. The stress inducible genes expressed during drought either encode functional proteins which directly protect plants against environmental stresses or they encode proteins that help in regulation of stress responsive genes^3,4^. Studies on response to drought stress have been performed in many arid region plants, including *Ziziphus nummularia*^5–7^ but the detailed study about the transcriptome involved in drought stress in *Z*. *nummularia* is scarce.

*Z*. *nummularia* (Burm.f.) Wight & Arn., commonly known as jhar ber or jhar beri belonging to the family Rhamnaceae, is a perennial herb native to tropical and dry regions, especially in the Thar Desert of western India, south-eastern Pakistan and southern Iran^8^. *Z*. *nummularia* is being utilized in a number of ways for the economic benefits. The leaves of this species are used as fodder^9,10^, the fruit is used as food^11–13^, particularly during food scarcity in many provinces of India. Since the shrub yields very hard wood, it is used to make tool handles, walking sticks and also for combustion purposes^14^. Over the years, the health benefits of Jhar ber have been extensively reported, including its antidiabetic, anti-inflammatory, antimicrobial, antioxidant, antitumor and immune system stimulant properties^15–17^. The anatomical characteristics like presence of high frequency of papilla on both surfaces, epidermis with thick outer wall and thick cuticle, and presence of stomata in crypts, place *Z*. *nummularia* in the xerophyte category of plants. As a xerophyte, *Z*. *nummularia* is bestowed with great tolerance to various abiotic stresses, such as drought, salinity and high temperature, which makes it an untapped non-model plant resource to uncover the mechanism underlying stress tolerance and to explore the candidate genes involved in stress tolerance^7^.

Only a few studies have demonstrated the molecular mechanism and gene expression patterns involved in the drought tolerance of Indian jujube^7^. The problem of studying the genetic basis of drought tolerance in *Z*. *nummularia* is further complicated by lack of sequence data, as evidenced by the availability of only 1,243 ESTs (till May 30^th^ 2017) in the GenBank database (https://www.ncbi.nlm.nih.gov/nucest/?term=ziziphus+nummularia). Therefore, it is imperative to carry out studies exploring the unique molecular adaptive strategies exhibited by this non-model plant species in response to drought stress.

Overall, the knowledge of *Ziziphus* genomics is in its infancy with limited genome-wide studies being available in the public domain. *Z*. *jujuba* (Chinese ber) represents the first and the only genome to be sequenced from the Rhamnaceae family^18^. The limited genomic information has further hampered the molecular breeding and SSR marker development. Molecular markers are robust tools to study the plant genomes and the genetic variations associated with particular phenotypes^19^. A number of molecular markers have been developed over the years and among them simple sequence repeats (SSRs) or microsatellite loci are preferred because they are simpler, reproducible, abundant, hyper variable, effective and co-dominant^20^. Currently, no SSRs are seemingly reported in *Z*. *nummularia*, though a few have been reported in other species like Z. *mauritiana*^21^, *Z*. *jujuba*^22,23^, and *Z*. *jujuba* var. *spinosa*^24^. The absence of high-throughput marker collection is a major bottleneck in conducting studies on population genetics and high density mapping in *Z*. *nummularia*. Therefore, the identification of robust SSR markers is of paramount importance for conducting such studies in *Z*. *nummularia*.

Of late, the Next-Generation Sequencing (NGS) of RNA has emerged as an excellent approach for isolation of novel genes and development of molecular markers like SSRs^25,26^, particularly, in the previously unannotated genomes^27,28^. In the present study, we have performed transcriptome sequencing of *Z*. *nummularia* under drought stress using 454 GS-FLX sequencing platform. The *de novo* assembly and annotation led to the identification of candidate genes involved in drought tolerance. The differential expression patterns of the drought-responsive genes have been validated by qRT-PCR analysis. This is the first report on transcriptome sequencing in *Z*. *nummularia*, the large-scale sequencing data generated will help in the discovery of novel genes associated with drought stress. The SSR markers identified shall assist in breeding studies on *Z*. *nummularia* and the genes identified in this study can be characterized and further deployed for developing drought-stress tolerant transgenic crops.

## Material and Methods

### Plant materials and stress conditions

The seeds of *Z*. *nummularia* were procured from the Central Institute for Arid Horticulture (ICAR-CIAH), Bikaner, India. After removing the hard coat, the seeds of *Z*. *nummularia* were sown in 15 cm diameter pots containing soilrite and allowed to grow in growth chambers at 30 °C with 16 h light/8 h dark photoperiod at the National Phytotron Facility, Indian Agricultural Research Institute, New Delhi, India. Four-week-old seedlings were used to initiate drought stress. Fifteen whole seedlings (3 for each hour of treatment) were treated with 30% Polyethylene glycol M.W. 6000 (PEG 6000) in 0.5x  Murashige and Skoog media (w/v) (Himedia, India) supplemented with 1 mM 2-(N-morpholino) ethanesulfonic acid (MES) (w/v) (Himedia, India) to introduce drought stress at 0.1 Mpa (osmotic potential). The seedlings (whole plant including roots, stem and leaves) were harvested after 6 h, 12 h, 24 h, 48 h and 72 h of treatment and three untreated (control) seedlings were also harvested simultaneously for further downstream processing. All the harvested seedlings were immediately frozen in liquid nitrogen and stored in a deep freezer (−80 °C) until use.

The osmotic potential was calculated as follows:

$${\rm{Osmotic}}\,{\rm{potential}}\,({\rm{\psi }}{\rm{\pi }})=-{\rm{CRT}}$$

where, C denotes the molar concentration of solute in molL^−1^, R is the universal gas constant (R = 8.314 kPa L/mol K) and T is the absolute temperature (i-e, experimental temperature 30 °C + absolute temperature).

### RNA extraction and transcriptome pyrosequencing

The total RNA from both treated and control seedlings was extracted using Spectrum^TM^ Plant Total RNA kit (Sigma-Aldrich, St. Louis, MO 63103, USA) as described by the manufacturer and DNA contamination was removed by on column DNase I Digestion set (Sigma-Aldrich). The integrity and concentration of RNA were checked by electrophoresis on 1.2% agarose/EtBr gel in 1X TAE buffer, UV spectrophotometry (Thermo scientific, Waltham, MA, USA) and a Bioanalyzer Chip RNA7500 series II (Agilent Technologies, USA). The mRNA was purified from 3 µg total RNA using Oligotex mRNA mini kit (Qiagen, Germany) as per manufacturer’s instructions. Equal concentrations of purified mRNA (200 ng) from both control and treated samples were used as template for random hexamer-primed synthesis of first strand cDNA, followed by double strand cDNA synthesis using cDNA Synthesis System Kit (Roche, Switzerland). The double strand cDNA was purified by PCR purification kit (Qiagen) and concentration of the purified cDNA was determined using Bioanalyzer 2100 (Agilent Technologies). The purified double strand cDNAs were sheared via nebulization into small fragments, followed by ligation to adapters using GS FLX Titanium Rapid Library MID Adaptors kit (Roche, Switzerland). The libraries were pooled before sequencing and loaded on to Titanium PicoTiter Plate followed by pyrosequencing on Roche GS-FLX454 Titanium platform.

### *De novo* assembly, annotation and functional characterization

The raw sequences generated from pyrosequencing were subjected to quality control analysis for removal of short-sized and poor quality reads (like adaptor sequences, reads containing poly N and sequences <50 bp) using FastQC tools v0.11.2 (http://www.bioinformatics.babraham.ac.uk/projects/fastqc/). The *de novo* assembly of high quality reads was performed using iAssembler v1.0 (beta) (http://bioinfo.bti.cornell.edu/tool/-iAssembler/). The assembled unigenes were annotated against non-redundant (NR) protein database through BLASTx with E-value cut-off of 10^−6^ (http://www.ncbi.nlm.nih.gov/). The functional categorizations were searched against TAIR (http://www.arabidopsis.org/) through BLASTx using threshold E-value value of 10^−6^. Gene ontology analysis of annotated transcripts was performed using Blast2GO version 2.8 (https://www.blast2go.com/)^29^ and the transcripts were divided into three groups, *viz*. biological process, molecular function and cellular component. The biochemical pathways of annotated transcripts were carried out by KEGG mapping^30^ using Blast2GO program.

### Mining of simple sequence repeats (SSRs)

The Perl script of MISA–MicroSAtellite identification tool (http://pgrc.ipk-gatersleben.de/misa/) was used for the identification of mono-nucleotide, di-nucleotide, tri-nucleotide, tetra-nucleotide, penta-nucleotide and hexa-nucleotide simple sequence repeats (SSRs) in the unigenes generated by sequencing. Ten contiguous repeats for mono-nucleotides, six contiguous repeats for di-nucleotides and five contiguous repeats each for tri-nucleotide, tetra-nucleotide, penta-nucleotide and hexa-nucleotides were considered as search criteria for SSR identification.

### Differential expression analysis of drought-responsive genes

In order to validate the robustness of the obtained transcriptome profile read analysis, 16 putative drought-responsive genes were selected and their expression patterns were studied at different intervals of PEG6000-induced drought stress by qRT-PCR analysis. Primers were designed by using Integrarted DNA technology (IDT) software. The description of the genes selected and their corresponding primer sequences are given in Table 1; the related BLAST annotations of these genes is provided in supplementary Table S1. The total RNA was isolated from control and stressed samples, as described above and cDNA was synthesized by SuperScript^®^III first strand synthesis system (Invitrogen, USA) using oligo (dT) primer. *Ziziphus* elongation factor1 *(Zjef1)* [GenBank: EU916202] gene was used as internal control^31^. The reaction mixtures of qRT-PCR were set up in a total volume of 10 µl containing 100 ng template cDNA, 200 nM of each primer and 1 µl of 10X KAPA SYBR FAST qPCR master mix buffer (KAPA Biosystems, USA). The qRT-PCR was carried out in LightCycler® 480 II (Roche) with thermal cycling conditions as: initial denaturation at 94 °C for 3 min, followed by 40 cycles at 94 °C for 30 sec, 60 °C for 30 sec and 72 °C for 30 sec. The Ct (threshold cycles) value were calculated by 2^−∆∆CT^ method as described by Padaria *et al*.^7^, where Ct values from three replicates were averaged and normalized with the Ct values of internal control *Zjef1* and specificity of the reactions was confirmed by melting curve. The statistical significance of the difference in relative expression of treated and control plants was calculated by Tukey’s multiple comparison test.

**Table 1:** Description of the putative drought responsive genes and their respective primer sequences used for qRT-PCR analysis.

Gene	Forward primer sequence (5′ to 3′)	Reverse primer sequence (5′ to 3′)
Cell wall-associated hydrolase	GTTCATCTTCGGCGCAA	CTCAGTGATAAAGGAGGTAGGG
Secretory carrier membrane protein	TTGGGAGATCATGCTTTGG	GCTGCCTCGGAAATACAT
Glycine-rich RNA-binding protein	GTCGTGAGGGAGGATACAA	CCACCATCACCGTATCCA
ATP synthase	CCCTACCCGTCATTGAAAC	CCGACGTTAATAGCAGGTC
Calcium-dependent protein kinase	GAACTCTGTGCTGGTTTGGA	CACGATGCATCACACCCATA
ABC transporter C family	CCTTAAGACATCCTGCTTCTTC	TGCTTCAACTCCAGCTCTA
Hydroxyproline-rich glycoprotein family protein	GGAATGTGGGTGATGGTGTAG	AGCGAGTCACCAGAAGAAAC
Late embryogenesis abundant protein	TCATGAGCCAAGAACAGC	TCGGCGCAATTGGTTTAG
Serine/threonine-protein phosphatase,	CCTGCAAACAAGGTGGTACT	CATTCTTCCTCAGGCTCTTCC
Asparagine synthetase	GCTAGGAGTGAAGATGGTTATC	CATACATATGCAGTGCCTTAATC
Auxin efflux carrier	CGTATTCCAGCCTCATTGG	GAAGAGCCATAAAGAGACCTAAA
Catalase	CCCAATTCCTTCTGCCATCT	AATCGCTCTTGCCTGTCTG
Heat shock protein 20	CTAAGGCCGAGAAGAAGGAAG	GAGGCACAGGAACAACTACA
*Wrky* transcription factor	TTCCTCCTGAGTCTCCAATCT	CCATCTGGGCAATACCTTTCT
Heat shock protein 70	GCAAATGGGTCTTGGTGAGT	CAACCGATTCAGGGCTTCAT
Chaperone protein *ClpB*3	CAACAATCAGAGAGGGAGAAGC	TCCGATAGACCTGCTCTTGA
*Ziziphus* elongation factor1	GCTGACTGTGCTGTTCTCATC	GACACCAAGAGTGAAAGCGAG

### Data availability

The high-quality sequencing reads obtained in this study were deposited to Short Read Archive (SRA) database of NCBI (http://trace.ncbi.nlm.nih.gov/Traces/sra/sra.cgi?view=studies) and were assigned the accession number SRX1118834.

## Results

### *De novo* assembly, annotation and Functional characterization

**Figure 1:**
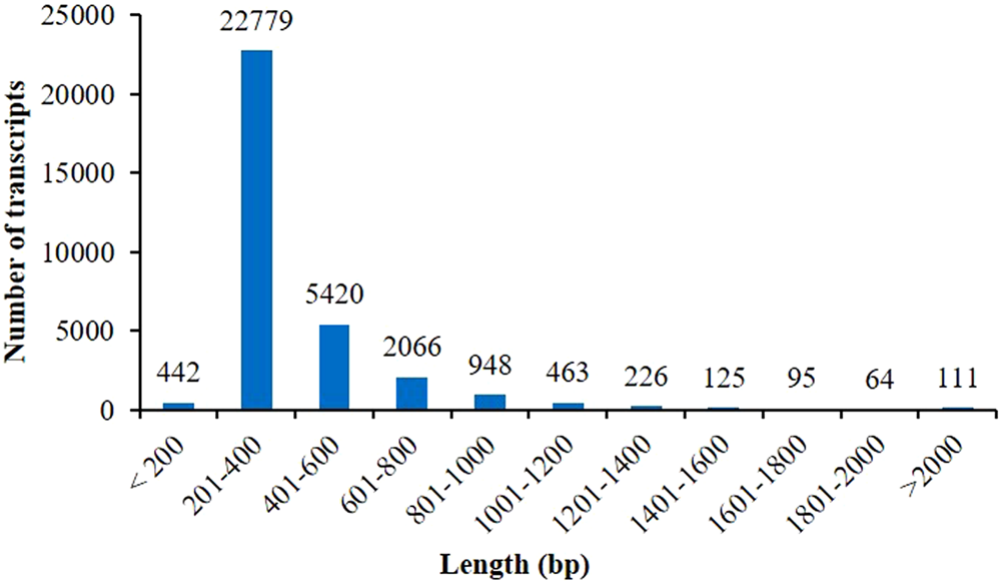
Sequence length distribution for assembled sequence reads.

To gain insights into the transcriptome profile of *Z*. *nummularia* in response to drought stress, a pooled cDNA library at different intervals of PEG 6000 induced drought stress was constructed and sequenced. A total of 367,176 raw reads were generated and after removing the low-quality reads, 351,872 (95.83% of all the reads) high quality (HQ) reads were used for subsequent analysis. Among these HQ reads, control (C) and stress (S) library contained 1,14,012 and 2,37,860 sequences, respectively (Table 2). The *de novo* assembly was carried out using iAssembler v1.0 (beta) tool with default or optimized parameters. A total of 351,872 high quality reads were assembled into 32,739 unigene**s** (Supplementary Table S1). The total size of transcripts including gaps was 1,34,76,223 bp. The length of unigenes ranged from 200 bp to 5,167 bp, with an average length of 412 bp, the mean transcript length and N50 values were 412 bp and 396 bp, respectively (Table 2). The nucleotide length of 442 transcripts (1.35%) was upto 200 bp, of 22,779 transcripts (69.57%) was 201–400 bp, of 5,420 transcripts (16.55%) was 401–600 bp, of 2,066 transcripts (6.31%) was 601–800 bp, of 948 transcripts (2.89%) was 801–1,000 bp, of 463 transcripts (1.41%) was 1,001–1,200 bp, of 226 transcripts (0.69%) was 1,201–1,400 bp, of 125 transcripts (0.38%) was 1,401–1,600 bp, of 95 transcripts (0.29%) was 1,601–1,800 bp, of 64 transcripts (0.19%) was 1,801–2,000 bp and of 111 transcripts (0.33%) was >2,001 bp (Fig. 1) with an average GC content of 42.93%.

**Table 2:** Summary of transcriptome sequencing and assembly in *Ziziphus nummularia*.

Statistics	Results
Raw reads from control library	1,18,386
Raw reads from stress library	2,48,790
Reads after trimming from C library	1,14,012
Reads after trimming from S library	2,37,860
High quality sequence used for assembly	3,51,872
Total number of unigenes	32,739
Size of transcripts including gaps	1,34,76,223 bp
Longest transcript	5,167 bp
Shortest transcript	200 bp
Mean transcript size	412 bp
Median transcript size	337 bp
N50 transcript length	396 bp
Transcript %GC	42.93

For annotation and functional characterization, non-redundant (NR) databases of NCBI and TAIR were used. All the unigenes were aligned with unigenes of other plant species using BLASTx program with an E-value cut-off of 10^-^^06^. Out of 32,739 unigenes annotated, 20,291 (61.97%) showed significant BLAST matches and it was observed that many unigenes (two or more) were annotated against the same protein. A total of 9,487 (28.97%) unigenes did not show any hits with the known protein, so they may be considered as putative novel unigenes. The number of annotated sequence showing similarity to *Ziziphus jujuba* was the largest (74.36%), followed by *Morus notabilis* (2%), *Glycine max* (1.58%), *Vitis vinifera* (1%) and *Citrus sinensis* (0.89%) (Supplementary Figure S1, Supplementary Table S2).

**Figure 2:**
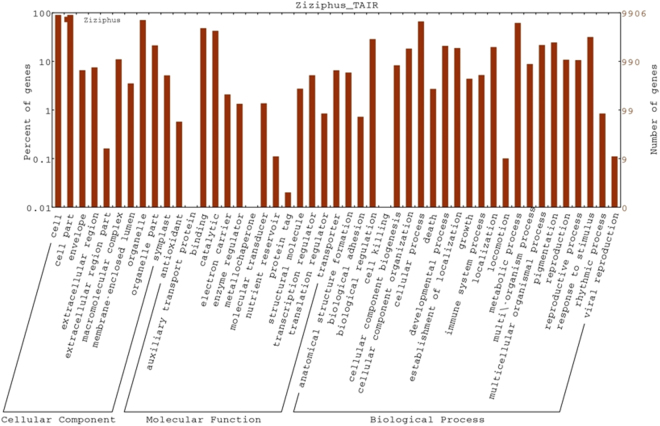
Histogram representing gene ontology (GO) classification of the assembled unigenes. The results are summarized in three main GO categories: cellular component, molecular function and biological process. The x-axis depicts the sub-categories of the unigenes, and the left and right y-axes represent the percentage and the number of unigenes, respectively.

Gene ontology assignments, functional annotation and categorization of assembled unigenes of *Z*. *nummularia* were carried out by using TAIR database. BLASTx results indicated that 20,345 of the 32,739 unigenes matched with a particular accession number or gene. Out of 20,345 unigenes, 9,906 unigenes were annotated to GO terms one or more times and they were categorized into 47 functional groups. All these functional groups were further categorized into three main categories of gene ontology classification *viz*. biological process, molecular function and cellular component. The biological process group was further classified into 23 sub-groups with the majority of unigenes being explicated in cellular (6,576, 66.4%) and metabolic process (6,124, 61.8%) categories (Fig. 2). The molecular function category was divided into 14 sub-groups with majority of the unigenes being delineated in binding activity (4,773, 48.2%) and catalytic activity (4,211, 42.5%) (Fig. 2). The cellular component category was classified into 10 sub-functional groups and unigenes under cell group were the most dominant (8,938, 90.2%) followed by organelle (7,011, 70.8%), organelle part (2,113, 21.3%), macromolecular complex (1093, 11.0%), extracellular region (746, 7.5%), envelope (652, 6.6%), membrane-enclosed lumen (349, 3.5%) and extracellular region (16, 0.2%) sub-groups (Fig. 2).

### KEGG pathway mapping

**Figure 3:**
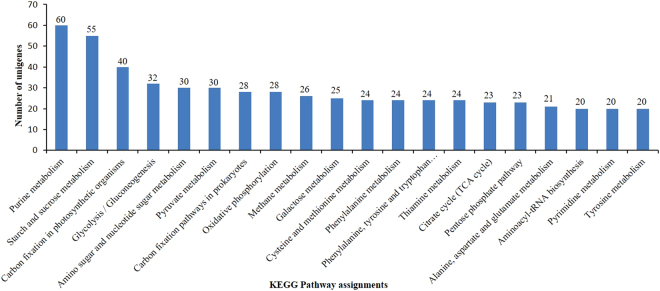
Histogram representations for the top twenty pathways of unigenes assigned based on the Kyoto Encyclopedia of Genes and Genomes (KEGG). The columns signify the number of unigenes in each subcategory.

The KEGG (Kyoto Encyclopedia of Genes and Genomes) pathway analysis revealed that out of 2,571 annotated unigenes, 1,140 had KEGG orthologs which were assigned to 114 pathways (Supplementary Table S3). The purine metabolism, starch and sucrose metabolism, carbon fixation in photosynthetic organisms, glycolysis/gluconeogenesis and amino sugar and nucleotide sugar metabolism pathways were the most representative with 60, 55, 40, 32 and 30 unique sequences, respectively. The top 20 KEGG pathway groups are represented in Fig. 3.

### Distribution and frequency of SSRs in the *Z*. *nummularia* transcriptome

Using MISA tool, a total of 32,739 sequences containing 1,34,76,223 bp were examined and 3,425 SSRs distributed in 2,813 sequences were identified. The EST-SSR frequency in the transcripome was observed at 8.59%. As per the classification criteria proposed by Weber^32^, the most abundant repeat motif was mono-nucleotide (1,453, 42.4%), followed by di-nucleotide (982, 28.67%), tri-nucleotide (874, 25.5%), tetra-nucleotide (80, 2.33%), hexa-nucleotide (21, 0.60%) and penta-nucleotide (15, 0.45%) (Table 3). 414 out of 3,425 SSRs were present in compound formation and the remaining 3,011 were observed to be the perfect SSRs. Among 3,011 perfect SSRs, 333 were found to be compound SSRs and the summarized details are in supplementary Table S4. The number of sequences having more than one SSR was 491. The frequencies of SSRs with different number of tandem repeats were calculated and it was observed that SSRs with five tandem repeats (24.54%) were the most abundant, followed by six tandem repeats (22.71%), >10 tandem repeats (21.9%), seven tandem repeats (13.89%), eight tandem repeats (8.67%), nine tandem repeats (8.26%) and 10 tandem repeats (6.69%) (Supplementary Table S5). Among the di-nucleotides repeats identified, AT/AT motif was the most predominant, constituting 25.30% of all the di-nucleotides characterized, which was followed by AG/CT (18.96%), AC/GT (5.22%) and CG/CG (0.30%) motifs. Among the tri-nucleotide repeats, AAG/CTT motif was the most predominant amounting to 14.09% of all the tri-nucleotides characterized, followed by AAT/ATT (9.22%), ACC/GGT (5.52%), ATC/ATG (4.56%), AGC/CTG (2.89%), AGG/CCT (2.78%), ACG/CGT (0.96%), CCG/CGG (0.76%) and ACT/AGT (0.50%) motifs. A total of 80 tetra-nucleotide repeat motifs were identified, among them AAAT/ATTT (2.43%) was found to be the most abundant. The number of penta-nucleotide and hexa-nucleotide motifs identified was 15 and 21, respectively, with AAAAT/ATTTT (0.45%) as the most predominant penta-nucleotide motif and AAAAAT/ATTTTT (0.20%) as the most frequent hexa-nucleotide motif. The frequencies of all the identified repeat motifs are summarized in supplementary Table S5.

**Table 3:** Frequency of different SSRs observed in *Ziziphus nummularia*.

Motif length (nucleotides)	Repeat numbers							Total motifs	Percentage in total (%)
	5	6	7	8	9	10	>10		
Mono-						458	995	1,453	42.42
Di-		224	145	115	129	103	266	982	28.67
Tri-	412	198	120	54	33	28	29	874	25.51
Tetra-	50	21	2	1	0	1	5	80	2.33
Penta-	8	2	4	1		0	0	15	0.43
Hexa-	14	3	3	0	1	0		21	0.61
**Total**								**3**,**425**	

### Differential expression analysis of genes involved in drought tolerance

**Figure 4:**
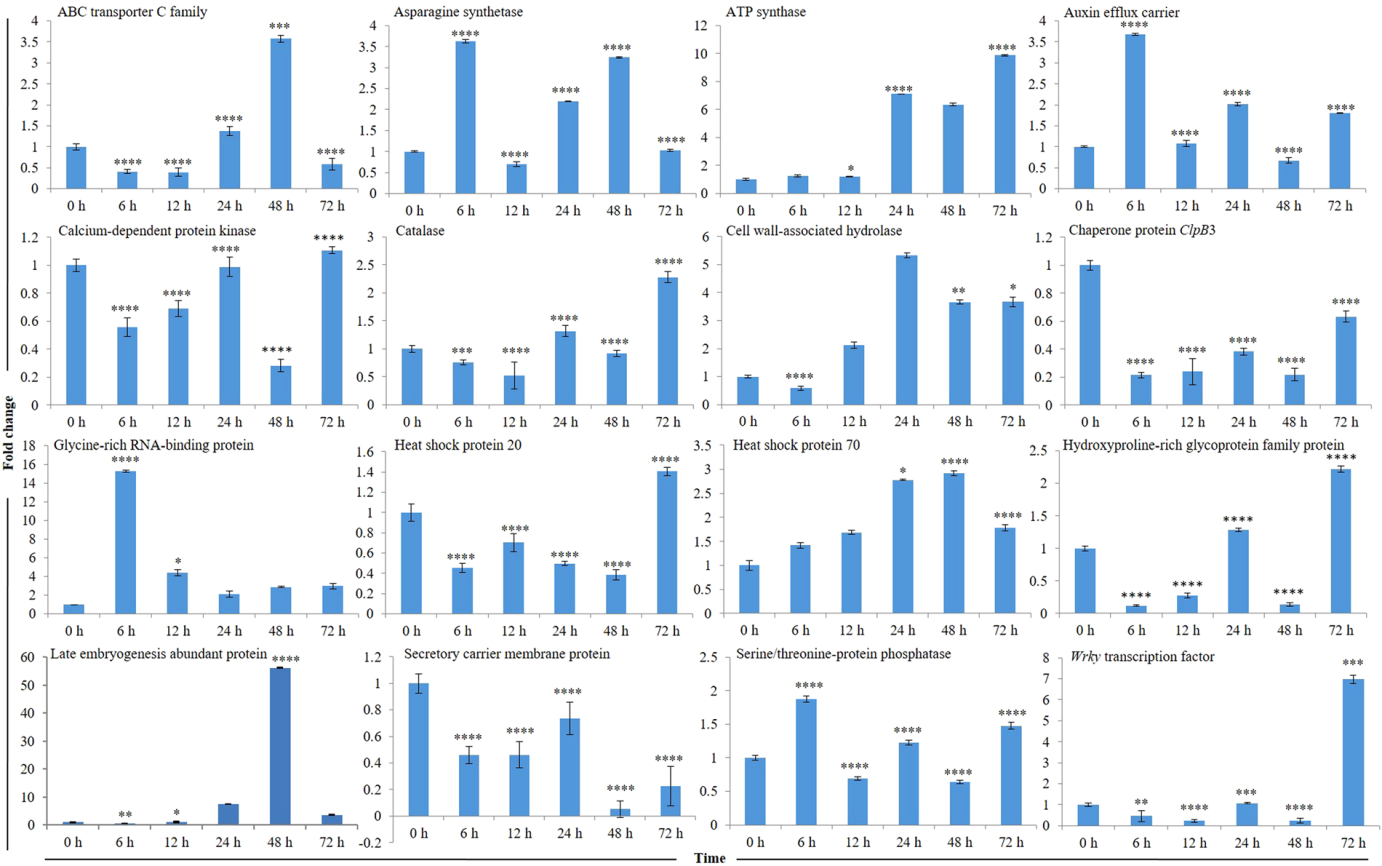
qRT-PCR expression profile of the putative drought responsive genes in *Z*. *nummularia*. Duration of PEG-induced drought stress (in hours) and Fold change in gene expression are shown on X and Y axes, respectively. *Ziziphus* elongation factor1 *(Zjef1)* was used as an internal control. The data represents mean values ± SE (n = 3) and vertical lines indicate calculated standard error. The asterisks designate statistical significance between the control and treated seedlings (*P < 0.05; **P < 0.01, ***P < 0.001 and ****P < 0.0001).

Based on the qRT-PCR data analysis, the selected genes displayed different expression profiles, with chaperone protein ClpB3 and secretory carrier membrane protein encoding genes showing significant down regulation compared to the control, and genes encoding calcium-dependent protein kinase and heat shock protein 20 showing expression at par with the control (Fig. 4). All other genes displayed significant up-regulation with time compared to the control, with *LEA* gene showing the maximum up-regulation (56.2-fold) at 48 h of PEG 6000 treatment (Fig. 4). The qRT-PCR expression patterns of all the candidate genes were in agreement with the transcriptome data, and therefore validated the results of the first *de novo* transcriptome data.

## Discussion

*Z*. *nummularia*, an inherently drought-tolerant non-model plant, is an important untapped genetic resource to study the genetic and metabolic control of adaptation to drought stress^7^. Next-generation sequencing technology (NGS) has been extensively used to study the model plant transcriptomes and due to accuracy, rapidity and low cost, its use has also been extended to unearth the unique genetic characteristics in the non-model plant systems^33^. The sequences generated were *de novo* assembled using iAssembler software. The iAssembler generates initial assemblies by MIRA and CAP3 assemblers and makes correction in two common types of transcriptome assembly errors: i) ESTs from different transcripts (mainly alternatively spliced transcripts or paralogs) are incorrectly assembled into same contigs; and ii) ESTs from same transcripts fail to be assembled together to deliver highly accurate *de novo* assemblies of EST sequences^34^. In spite of the development of several bioinformatics tools for data assembly and analysis, *de novo* assembly of short reads without a reference genome, is very challenging task^35,36^. It is very important to choose a suitable assembler and parameters to assemble large-scale ESTs into consensus sequences with significantly high accuracy.

In the present study, the average GC content of *Z*. *nummularia* transcripts was observed to be 42.93% which is in agreement with the previously reported value in other dicots *viz*., 44.6% for *Picrorhiza kurrooa*^37^, 33.41% for genome of *Z*. *jujuba*^18^, and 41.0% for *Nicotiana benthamiana*^38^. The GC content represents the stability of DNA as well as of the genes and genomic composition including evolution, gene structure and regulation^39^, and the changing GC content may reveal the adaptability to different climatic conditions.

The functional annotation results showed that a total of 23,984 (73.25%) unigenes have significant BLAST matches, which is higher than the previously reported data, 54.9% in bamboo^40^, and 54.31% in *Ziziphus jujuba*^5^. This higher value indicates the extensive coverage of the *Z*. *nummularia* transcriptome in this study. A total of 8,112 (24.77%) unigenes did not match with any of the known proteins, this is a common feature associated with *de novo* sequencing and varying percentages like 45.73%, 40.70%, 62.48%, 42.78% by Dang *et al*.^41^, Long *et al*.^42^, Wu *et al*.^43^ and Ma *et al*.^6^, respectively of such unigenes has been reported previously. The presence of these un-identified unigenes can be partly ascribed to the dearth of genome and EST information of *Z*. *nummularia* in the public domain and also to the short read length commonly associated with the next-generation sequencing technology^6^. The gene ontology results revealed that a large number of unigenes were involved in cellular process, metabolic process and response to stimulus (Fig. 2). The functional GO results indicated the dominance of gene regulation, signal transduction and enzymatically active processes in transcriptome, as maximum unigenes were found to be belonging to the binding activity, catalytic activity, transport activity and transcription regulator activity categories (Fig. 2). The various categories identified by the GO analysis classified the unigenes as, those involved in response to abiotic and biotic stimulus. Among these, the unigenes were further found to be involved in heat acclimation, cellular response to hydrogen peroxide, osmotic stress, oxidative stress, unfolded protein, water deprivation, drought recovery, endoplasmic reticulum unfolded protein response, hydrogen peroxide catabolic process, starvation, osmosensory signaling pathway, priming of cellular response to stress and cation stress. All these categories are reported to participate in drought tolerance mechanisms^44,45^. Drought tolerance mechanism acts in combination with drought avoidance and tolerance periods; it also involves many genes that play a major role in osmotic and redox homeostasis processes and participate in the readjustment process. Some of the unigene categories identified were found to be involved in response to salt, cold and heat stresses, hyperosmotic response, and hyperosmotic salinity response, indicating that various abiotic stresses follow multiple pathways for stress perception and signaling, which cross-talk at various points^46^. These results suggested that transcriptome sequencing was successful in collecting the information about cytosolic response to drought stress in *Z*. *nummularia* and it would contribute to our understanding about the drought tolerance mechanism in *Z*. *nummularia*.

The KEGG pathway mapping resulted in the identification of 114 biological pathways from 1,140 annotated unigenes (Fig. 3, Supplementary Table S3). Li *et al*.^5^ reported 16,693 *Z*. *jujuba* annotated unigenes, consisting of 6,485 isotigs and 10,117 singletons, which were assigned to 319 KEGG pathways. Some of the pathways, identified in our study, that are involved in biosynthesis of genes responsive to drought stress are purine metabolism^47^, starch and sucrose metabolism^48^, fructose and mannose metabolism^49^, arginine and proline metabolism^50^, aminobenzoate degradation^43^, thiamine metabolism^51^, and phenylalanine metabolism^52^. These analyses showed that the diversity and variation in *Z*. *nummularia* genome provide precious information for investigating specific processes, functions and pathways and thereby making possible the discovery of novel genes which are responsive to drought stress in *Z*. *nummularia*.

In transcriptome of *Z*. *nummularia*, a total of 3,425 SSRs were identified with a frequency of 8.59%, which is lower than that observed in the fruit transcriptome of the related species *Z*. *jujuba* (10.1%)^5^, and floral transcriptome of *Z*. *celata* (17%)^53^. A total of 3,425 SSRs were recognised in 2,813 sequences, with one SSR observed for every 3.93 kb of the analysed sequences. Previously, one SSR locus for every 0.94 kb (kb/SSR) was detected in *Triticum aestivum*^54^, 3.87 kb/SSR in *Z*. *jujuba*^5^, 3.55 kb/SSR in *Camellia sinensis*^55^, 6.22 kb/SSR in *Arachis hypogaea*^56^, 6.69 kb/SSR in *Epimedium sagittatum*^57^, and 17.56 kb/SSR for *Cymbidium ensifolium*^58^ transcriptomes. The incidence of SSRs in plant genomes is governed by the repeat length and the standard used for searching SSRs in databases^59^. Moreover, the depth of sequencing coverage also influences the detection rate and since 454-pyrosequencing gives less depth compared to other contemporary sequencing platforms, the detection could be low in the present case. Among all the nucleotide repeat motifs, AT/AT, AG/CT and AAG/CTT were the most abundant SSR motifs and CG/CG was the least frequent. Our results are consistent with those observed in *Sesamum indicum*^60^, *Epimedium sagittatum*^57^, and other plant species^61,62^. These observations suggest that the transcriptome sequencing data is an ideal resource for mining the SSRs in *Z*. *nummularia* and its cross transferability will help in studying different species of this family.

Drought tolerance is a complex trait and various adaptive mechanisms are involved at multiple levels like cellular, molecular and morphological to cope with water-deficit conditions. In our study, a large number of drought-responsive genes were obtained in the transcriptome of *Z*. *nummularia* and their expression patterns were studied by qRT-PCR analysis. Among them, the cell wall associated hydrolase gene has been reported to play an important role in accumulation of compatible extra-cellular solutes through altering cell wall extensibility and other cell wall modifications under drought and salt stress. High expression levels of the cell wall associated hydrolase gene was reported in *Ammopiptanthus mongolicus* and may help the specific structural adaptations to cope with severe drought in deserts^63^. Glycine-rich RNA-binding protein harboring RNA chaperon activity has importance in RNA metabolism to prevent the adverse effect of environmental stresses on crop yield. Transgenic rice over-expressing *Arabidopsis thaliana* glycine-rich RBP (*At*GRP or *At*GRP7) has shown much higher stress-recovery rates and grain yields in comparison to the wild-type plants under drought stress conditions and has confirmed the importance of post-transcriptional regulation of RNA metabolism in plant under abiotic stress conditions^64^. ABC transporter gene is a membrane-intrinsic gene that has shown high expression level and plays an important role in physiological processes to adapt the plant in changing environments and cope with biotic and abiotic stresses^65^. Based on the qRT PCR analysis, the maximum upregulation was observed for *Lea* gene (Fig. 4), many studies have reported that over-expression of LEA proteins improve abiotic stress tolerance in transgenic plants. For example, the expression of barley LEA protein (HVA1) conferred drought tolerance in transgenic wheat and rice^66,67^, and wheat LEA proteins PMA80 and PMA1959 enhanced dehydration tolerance in transgenic rice^68^. The involvement of WRKY proteins in various abiotic stresses, such as salinity, drought, and cold has been reported previously^69,70^. *Wrky* gene from *T*. *aestivum* and *Oryza sativa* have been identified to play an important role in drought tolerance mechanism^71,72^. The enzyme catalase is a main component of anti-oxidative machinery^73^ and its expression and activities are always triggered by the environmental stresses^74–76^. The upregulation of catalase gene in *Z*. *nummularia* at 72 h has suggested that abiotic stresses enhance the transcription of catalase and subsequently control homeostasis in plant cell as described earlier^77^. The qRT-PCR expression patterns of all the candidate genes were found in agreement with the transcriptome data, therefore, the high-throughput RNA sequencing data generated could be considered fairly conclusive.

### Conclusion

*Z*. *nummularia* is categorized as a xerophyte plant species with inherent tolerance to drought stress and hence is a rich genetic resource to investigate drought-responsive genes and mechanism of drought tolerance. In this study, we have used a combination of *de novo* transcriptome sequencing, differential gene expression profiling and SSR marker identification to perform comprehensive analysis of the mechanism underlying drought stress and also for identification of drought-responsive genes in *Z*. *nummularia*. Computational analysis has confirmed that the maximum portion of *Z*. *nummularia* transcriptome is sequenced, and numerous genes and pathways are involved in tolerance to drought stress. The maximum number of ESTs showing similarity to the known databases and a substantial number of ESTs remaining un-identified do endorse the discovery of putative novel genes from the transcriptome data. Various SSR molecular markers have been characterized, which shall be useful in genotyping and breeding studies in *Ziziphus* and related species. The data generated can serve as a significant resource for bioprospecting drought-responsive candidate genes. Our findings will complement the available genomic resources and shall further provide the momentum to the ongoing efforts to ameliorate drought tolerance in crop plants.

## Electronic supplementary material

Supplementary Figure S1

Supplementary Dataset 1

Supplementary Dataset 2

Supplementary Dataset 3

Supplementary Dataset 4

Supplementary Dataset 5
