# Supplementary material for: Transcriptome sequence analysis and mining of SSRs in Jhar Ber (Ziziphus nummularia (Burm.f.) Wight & Arn) under drought stress
Source: Sci Rep. 2018 Feb 5;8:2406. doi: 10.1038/s41598-018-20548-1 (PMC5799245; doi:10.1038/s41598-018-20548-1)

Transcriptome sequence analysis and mining of SSRs in Jhar Ber (*Ziziphus nummularia (Burm.f.) Wight & Arn*) under drought stress

Radha Yadav[1†], Showkat Ahmad Lone[1, 2†], Kishor Gaikwad[1], Nagendra Kumar Singh[1] and Jasdeep Chatrath Padaria[1*]

Figure S1. Species distribution of top hits in transcriptome assembly

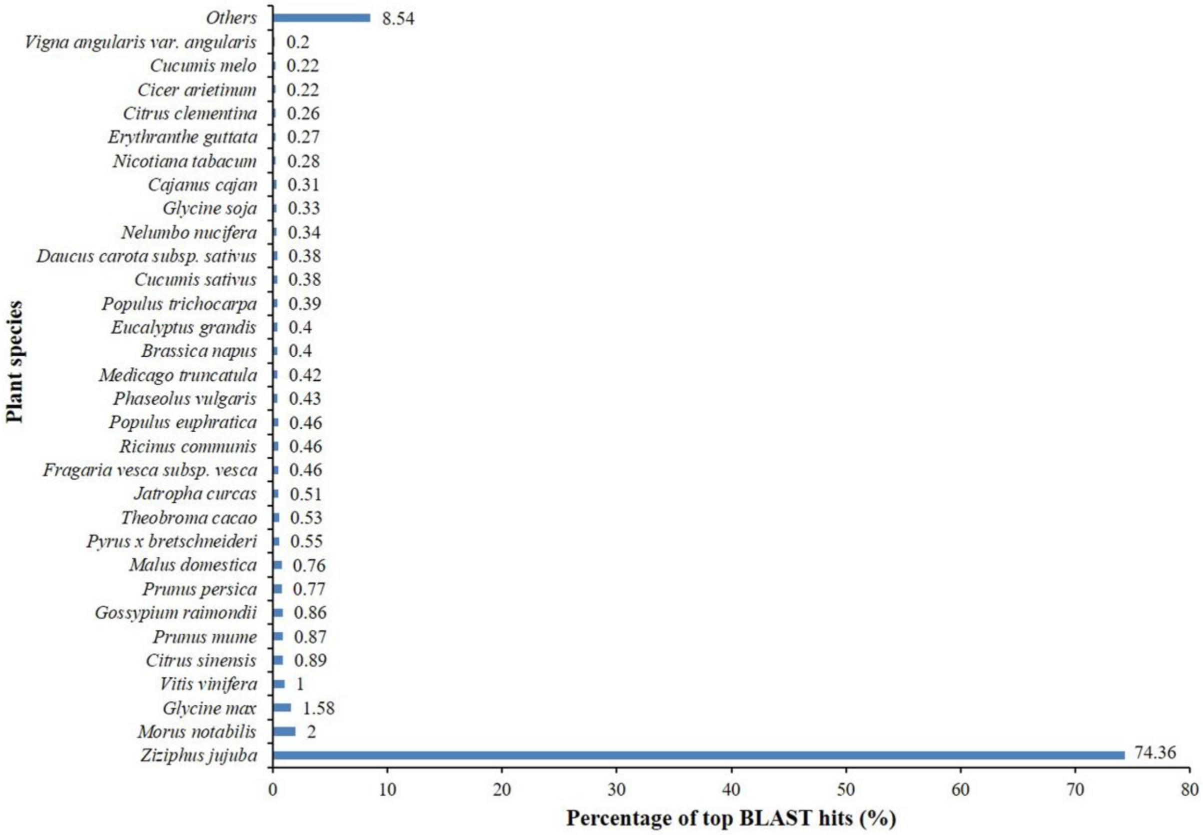

Supplement: Supplementary file 1 — Supplementary Figure S1 [file 41598_2018_20548_MOESM1_ESM.pdf]
